# A Longitudinal Study of Urinary Phthalate Excretion in 58 Full-Term and 67 Preterm Infants from Birth through 14 Months

**DOI:** 10.1289/ehp.1307569

**Published:** 2014-05-30

**Authors:** Hanne Frederiksen, Tanja Kuiri-Hänninen, Katharina M. Main, Leo Dunkel, Ulla Sankilampi

**Affiliations:** 1 University Department of Growth and Reproduction, Rigshospitalet, Faculty of Health Sciences, Copenhagen, Denmark; 2 Department of Pediatrics, School of Medicine, University of Eastern Finland, Kuopio, Finland; 3 Department of Pediatrics, Kuopio University Hospital, Kuopio, Finland; 4 William Harvey Research Institute, Barts and the London, School of Medicine and Dentistry, Queen Mary University of London, London, United Kingdom

## Abstract

Background: Some phthalates have shown antiandrogenic effects in rat offspring. Premature infants may be exposed to high amounts of specific phthalates during hospitalization, and thus are potentially at risk.

Objective: We evaluated longitudinal phthalate exposure and metabolism in full-term (FT) and preterm (PT) infants.

Methods: Fifty-eight FT and 67 PT (gestational age, 24.7–36.6 weeks) infants were recruited at birth and followed until 14 months (nine times). Urinary concentrations of metabolites of diethyl phthalate (DEP), dibutyl phthalate isomers (DiBP and DnBP), butylbenzyl phthalate (BBzP), di(2-ethylhexyl) phthalate (DEHP), and diisononyl phthalate (DiNP) were measured in 894 samples. Daily intake and a hazard index for antiandrogenic effects were estimated, and excretion patterns of DEHP and DiNP metabolites were analyzed.

Results: Metabolites of BBzP, DiNP, and DEHP were 5–50 times higher at day 7 (D7) and month 1 (M1) in PT than in FT infants. Thereafter, metabolite concentrations were similar between the two groups. The estimated hazard index for combined DiBP, DnBP, BBzP, and DEHP exposures 7 days after birth exceeded the antiandrogenic threshold in > 80% of PT and > 30% of FT infants, and after M2, in 30% of all infants. The excretion pattern of DEHP and DiNP metabolites changed with age.

Conclusion: Most PT infants and approximately one-third of healthy FT newborns were exposed to phthalates during early life at a potentially harmful level according to the European Food Safety Authority’s recommended limits of daily exposure. Changes in the relative proportions of secondary phthalate metabolites over time were consistent with maturation of infant metabolic pathways during the first year of life. Further research is needed on the health effects of phthalate exposures and the influence of changes in metabolic capacity in neonates and infants.

Citation: Frederiksen H, Kuiri-Hänninen T, Main KM, Dunkel L, Sankilampi U. 2014. A longitudinal study of urinary phthalate excretion in 58 full-term and 67 preterm infants from birth through 14 months. Environ Health Perspect 122:998–1005; http://dx.doi.org/10.1289/ehp.1307569

## Introduction

Phthalates are widely used as plasticizers in, for example, toys, cosmetics, food packaging, medical equipment, and building materials (Wittassek et al. 2011). Some of the most commonly used phthalates have been shown to cause outcomes in rats consistent with antiandrogenic effects, such as impaired spermatogenesis, undescended testes (cryptorchidism), and reduced anogenital distance (Borch et al. 2006; Foster 2006; Howdeshell et al. 2007).

Associations of early phthalate exposures with allergic asthma and reproductive and behavioral outcomes suggest that the fetus, neonate, and infant may be particularly vulnerable to endocrine-disrupting effects of these compounds (Bergman et al. 2012; Bornehag and Nanberg 2010; Main et al. 2006; Miodovnik et al. 2011; Swan et al. 2005, 2010; Whyatt et al. 2012). Despite these concerns, data on phthalate exposure levels during this period of life are sparse (Adibi et al. 2008; Enke et al. 2013; Sathyanarayana et al. 2008a, 2008b). Some studies have reported extremely high exposure to di(2-ethylhexyl) phthalate (DEHP) during hospitalization in preterm children (Silva et al. 2006; Su et al. 2012; Weuve et al. 2006), suggesting a risk of adverse health effects in this group as gonadal development is not completed (Rey and Josso 2013) and detoxification processes are still immature (Coughtrie et al. 1988; Tan et al. 1990).

In the present longitudinal study, we investigated urinary phthalate exposure from birth to 14 months of age in a group of full-term (FT) and preterm (PT) infants, covering periods of hospitalization and home stay. We also assessed whether these exposure levels exceeded European Food Safety Authorities (EFSA) guidelines for tolerable daily intakes (EFSA 2005a, 2005b, 2005c).

## Materials and Methods

*Study population*. As part of the Finnish Minipuberty study, a total of 173 mothers either with a normal singleton pregnancy or with a threat of premature delivery before 37.0 weeks of gestation were recruited in consecutive order inside these cohorts between August 2006 and March 2008 at Kuopio University Hospital, Finland. After delivery, infants of these mothers were recruited for the study, which included clinical examinations and sample collection at child’s age of 1–3 days (D1–3), 7 days (D7), and 1, 2, 3, 4, 5, and 6 months (M1–M6), and finally at the corrected age of 14 months [M14; 14 months from the expected date of delivery, approximately 100 weeks of postmenstrual (PM) age, where PM age was defined as sum of gestational age at birth plus postnatal age at examination to signify the “true” biological age of the infant]. Altogether, 125 children of 113 mothers completed the follow-up, and these included 58 FT (29 boys) and 67 PT (33 boys) infants (Table 1). A total of 894 urine samples were obtained at 1,067 visits. Two FT infants were twin sisters; nine PT boys and 11 PT girls were twins, and three PT girls were triplets. FT infants stayed at the hospital 2–18 days (mean, 4.3) while PT infants were hospitalized 3–156 days (mean, 39.6), up to 38–42 PM weeks (Kuiri-Hänninen et al. 2011a, 2011b).

**Table 1:** Birth characteristics: *n* = 125 [*n* (%) or mean ± SD (range)].

	FT infants	PT infants
Boys
*n* (%)	29 (50.0)	33 (49.3)
Gestational weeks	39.8 ± 1.37 (37.1–42.1)	31.8 ± 3.26 (24.7–36.6)
Birth weight (g)	3,274 ± 741 (1,910–4,420)	1,693 ± 548 (550–2,850)
Birth length (cm)	49.1 ± 2.88 (42–53)	41.3 ± 4.6 (30–48)
Days at hospital	4.9 ± 4.16 (2–18)	42.9 ± 32.0 (7–131)
Girls
*n* (%)	29 (50.0)	34 (50.7)
Gestational weeks	39.5 ± 1.33 (37.0–41.7)	32.9 ± 3.03 (24.7–36.7)
Birth weight (g)	3,373 ± 630 (2,070–4,750)	1,765 ± 522 (530–2,720)
Birth length (cm)	49.2 ± 2.61 (44–54)	41.8 ± 4.27 (29–47)
Days at hospital	3.8 ± 2.26 (2–12)	36.4 ± 31.4 (3–156)

*Ethics*. Both parents gave informed consent at initial recruitment and at follow-up at M14. The study was approved by the Ethics Committee of the Northern-Savo Health Care District.

*Sample collection*. Spot urine samples were collected in polyethylene urine collection bags (Paediatric Urine Collector, Unomedical, Lejre, Denmark, and U-Bag Pediatric, Mabis Healthcare, Waukegan, IL, USA) or by clean catch into polystyrene plastic cups (Econo Plastic Container, Huhtamäki OyJ, Finland) at every visit, and transported in 10-mL polypropene vials (Linkoputki, Mekalasi Oy, Finland). Urinary creatinine was measured by an enzymatic method before storage at –70°C in 3-mL polypropene vials (Mekamini, Mekalasi Oy, Finland).

*Chemical analysis*. Twelve primary and secondary metabolites of six phthalate diesters [diethyl phthalate (DEP), diisobutyl phthalate (DiBP), di-*n*-butyl phthalate (DnBP), butylbenzyl phthalate (BBzP), DEHP, and diisononyl phthalate (DiNP)] were analyzed. The total (sum of free and conjugated) content of monoethyl phthalate (MEP), monoisobutyl phthalate (MiBP), mono-*n*-butyl phthalate (MnBP), monobenzyl phthalate (MBzP), mono(2-ethylhexyl) phthalate (MEHP), mono(2-ethyl-5-hydroxyhexyl) phthalate (MEHHP), mono(2-ethyl-5-oxohexyl) phthalate (MEOHP), mono(2-ethyl-5-carboxypentyl) phthalate (MECPP), monoisononyl phthalate (MiNP), mono(hydroxyisononyl) phthalate (MHiNP), mono(oxoisononyl) phthalate (MOiNP), and mono(carboxyisooctyl) phthalate (MCiOP) was analyzed by isotope dilution liquid chromatography tandem mass spectrometry with preceding enzymatic deconjugation followed by automatic solid phase extraction. The method details have been described previously (Frederiksen et al. 2010). Samples were analyzed in 22 batches during 8 weeks. Each batch included standards, 40 samples, two blanks, two pooled urine control samples, and two pooled urine control samples spiked with standards to an added concentration of 10 ng/mL. The recovery was > 90% for all analytes and the interday variation, expressed as relative standard deviation (RSD), was < 3% for all analytes except MiBP (7%) and MnBP (9%). Limits of detection (LOD) are shown in Table 2.

**Table 2:** Urinary phthalate metabolite concentrations (ng/mL) in serial samples from birth to 14 months of age in 58 FT (*n* = 432 samples) and 67 PT (*n* = 462 samples) infants.

Phthalate metabolite	LOD	FT							PT						
		*n* > LOD	Percent > LOD	Min	10th	Median	90th	Max	*n* > LOD	Percent > LOD	Min	10th	Median	90th	Max
MEP	0.53	432	100.0	0.82	3.46	10.7	49.1	486	462	100.0	0.71	3.51	11.9	44.0	190
MiBP	1.43	431	99.8	< LOD	7.45	21.0	62.3	337	462	100.0	3.21	8.40	23.3	92.4	5,648
MnBP	1.10	431	99.8	< LOD	4.96	15.2	51.3	156	462	100.0	1.43	5.89	15.5	47.3	6,533
MBzP	1.14	432	100.0	1.85	6.66	24.6	118.0	1,985	462	100.0	2.13	8.66	43.1	244.0	1,155
MEHP	0.14	329	76.2	< LOD	< LOD	0.44	2.11	849	396	85.7	< LOD	< LOD	0.99	27.4	1,379
MEHHP	0.91	407	94.2	< LOD	1.27	5.01	26.0	480	451	97.6	< LOD	1.63	8.62	163.0	6,934
MEOHP	0.67	414	95.8	< LOD	1.06	3.90	15.3	351	452	97.8	< LOD	1.50	6.12	86.4	4,010
MECPP	0.55	432	100.0	0.75	3.74	10.7	40.2	1,366	462	100.0	0.81	5.85	17.9	668.0	26,011
ΣDEHPm					8.83	26.5	109.0	4,032				12.3	45.3	1284.0	46,488
MiNP	0.61	11	2.5	< LOD	< LOD	< LOD	0.18	2.88	49.0	10.6	< LOD	< LOD	< LOD	0.65	93.0
MHiNP	0.26	212	49.1	< LOD	< LOD	< LOD	2.16	27.1	312	67.5	< LOD	< LOD	0.61	6.46	135
MOiNP	0.25	200	46.3	< LOD	< LOD	< LOD	1.33	10.4	301	65.2	< LOD	< LOD	0.47	4.92	125
MCiOP	0.11	427	98.8	< LOD	0.29	1.32	5.40	58.2	458	99.1	< LOD	0.50	2.37	29.8	493
ΣDiNPm						2.36	11.6	107					4.74	62.7	1,035
Abbreviations: ΣDEHPm, the sum of DEHP metabolites; ΣDiNPm, the sum of DiNP metabolites; LOD, limit of detection; Max, maximum; Min, minimum. 10th and 90th are percentiles.															

*Statistics.* We measured one hydroxylated monoester and three oxidized secondary metabolites of DEHP and DiNP, respectively. To simplify data analysis, we calculated the molar sum of DEHP metabolites (MEHP, MEHHP, MEOHP, and MECPP) and DiNP metabolites (MiNP, MHiNP, MOiNP, and MCiOP) by multiplying the molar sums with the molecular weights of DEHP and DiNP, respectively (ΣDEHPm and ΣDiNPm in nanograms per milliliter, respectively).

Medians, percentiles, and ranges (minimum–maximum) of all measured phthalate concentrations were computed. All urinary metabolite concentrations < LOD were set to 0.0 except for the analyses of intraclass correlation coefficients (ICCs) or if ln-transformation was used; in these cases, data < LOD were set to LOD divided by the square root of 2.

We also provide descriptive statistics for samples after adjustment for urinary creatinine to account for dilution. However, because a child’s urinary creatinine 1–3 days after birth reflects maternal creatinine (Matos et al. 1998), we excluded samples collected on D1–3 from this analysis.

Within- and between-person variance as well as ICCs were calculated. The ICC takes a value between 0 and 1 and reflects the relationships between the within- and between-subject variance. ICCs were classified as weak (< 0.4), moderate (0.4–0.6), or good (> 0.6) (Philippat et al. 2013).

For each child *i* we estimated the daily intake (*DI*) (micrograms per kilogram per day) of each phthalate diester *p* at sampling time *t* as:

*DI_pit_* = [Σ*^^n^^_k_*_= 1_ (*UE_kit_*/*MW_k_*)] × *MW_p_* × *CE*_smoothed_/(*FUE_p_* × *BW_it_*), [1]

where *UE_kit_* represents the creatinine-adjusted urinary concentration (micrograms per gram creatinine) of metabolite *k* in child *i* at time *t*, *MW_k_* is the molecular mass of metabolite *k* (micrograms per micromole), *MW_p_* is the molecular mass of diester *p* (micrograms per micromole), and *CE*_smoothed_ is the estimated average 24-hr urinary creatinine excretion (grams per day) based on a study of newborns at 28–42 weeks of gestation (Al-Dahhan et al. 1988). *FUE_p_* is the estimated fraction of diester *p* excreted in urine, and *BW_it_* is the body weight (kilograms) of child *i* at time *t*. We assumed the following values of FUE*_p_* based on studies of adults after oral intake of deuterium labeled phthalate diesters: 84% of DnBP excreted as MnBP, 70.3% of DiBP as MiBP, 70% of BBzP as MBzP, 45.3% of DEHP as DEHP metabolites (MEHP, MEHHP, MEOHP, and MECPP), and 30% of DiNP as DiNP metabolites (MiNP, MHiNP, MOiNP, and MCiOP) (Anderson et al. 2001, 2011; Koch et al. 2012). For DEP we assumed an FUE of 69% (excreted as MEP) based on previous estimate for DnBP excretion. This estimate has often been used, because a human kinetic study on DEP is missing (Koch and Calafat 2009). To obtain a single median and selected percentile estimates for each phthalate at each sampling time (D7 through M14) according to PT or FT birth, estimates of DI for each child at time *t* were calculated as centiles. DI was not estimated for infants at time point D1–3, because creatinine is known to be elevated due to maternal contamination (Matos et al. 1998).

We estimated a hazard quotient (HQ) for phthalate diesters based on tolerable DI (TDI) values established by the EFSA (2005a, 2005b, 2005c). TDI values for DnBP [10 μg/kg/day, ~ lowest observed adverse effect level (LOAEL) with an uncertainty factor of 200], BBzP [500 μg/kg/day, ~ no observed adverse effect level (NOAEL) with an uncertainty factor of 100], and DEHP (50 μg/kg/day, ~ NOAEL with an uncertainty factor of 100) are based on experimental animal studies. Because DiBP has shown effects similar to those of DnBP (Borch et al. 2006), TDI for DnBP was used to calculate HQ for DiBP. We divided estimated DIs by their corresponding TDI to derive HQs for phthalate diesters with evidence of antiandrogenic effects—DiBP, DnBP, BBzP, and DEHP—and combined the HQ estimates for these compounds to derive a single estimated hazard index (HI) for each infant (Koch et al. 2011; Soeborg et al. 2012):

*HI_t_* = Σ*^^n^^_p_*_= 1_ (*DI_pt_*/*TDI_p_*), [2]

where *DI_pt_* is the estimated average daily intake of diester *p* (micrograms per kilogram per day) at time *t*, and *TDI_p_* is the EFSA guideline value (micrograms per kilogram per day) for diester *p* (as indicated above). *HI* = 1 is the antiandrogenic threshold, and a level > 1 indicates a potential risk of anti-androgenic effects.

We used mixed-models analysis for statistical testing to account for the correlation structure because of the repeated measurements and multiple births in the cohort. We derived between- and within-group comparisons of phthalate metabolite concentrations, and estimated differences according to sex, hospitalization at the time of sampling (yes/no), breastfeeding (none, partial, or full), and child’s weight at sampling. Data were analyzed according to calendar age (i.e., time from birth) and, to account for the immaturity in the PT infants, according to PM age (defined as sum of gestational age at birth plus postnatal age at examination) categorized into intervals (30.1–34.0, 34.1–38.0, 38.1–42.0, 42.1–46.0, 46.1–50.0, 50.1–54.0, 54.1–58.0, 58.1–62.0, 62.1–68.5, and 96–106 PM weeks) corresponding to sampling times at D1–3, D7, M1–M6, and the corrected age of M14, respectively. Birth group (PT or FT), time point (or PM age category), sex, breastfeeding, hospitalization, and weight were modeled as fixed effects, whereas subject and an indicator for multiple births were modeled as random effects. Associations between the concentrations of different phthalate metabolites and estimated DI were also analyzed using the mixed model. We report standardized coefficients of natural log (ln)–transformed phthalate metabolite concentrations, which are interpreted as correlation coefficients. *p*-Values < 0.05 were considered statistically significant. SPSS software version 19.0 (SPSS Inc., IBM, Chicago, IL, USA) was used for statistical analyses.

## Results

*Phthalate concentrations*. At least one metabolite of each of the six phthalates was detectable in virtually all urine samples of both FT and PT infants, indicating that all infants were exposed to DEP, BBzP, and DEHP, and that almost all infants were exposed to DiBP, DnBP, and DiNP at all time points. Only in one single urine sample were metabolites of DiBP or DnBP not detectable, whereas five urine samples did not contain measurable concentrations of any of the DiNP metabolites (Table 2). ΣDEHPm had the highest median concentration in both groups, followed by MBzP, MiBP, MnBP, MEP, and ΣDiNPm. Except for MEP and MnBP, PT infants had significantly higher median concentrations than did FT infants for all metabolites (*p <* 0.05 for MiBP; otherwise *p* < 0.001). Creatinine-adjusted phthalate metabolite concentrations were about 10 times higher than measured concentrations, with similar differences between PT and FT infants as in the unadjusted values (see Supplemental Material, Table S1).

**Figure 1:**
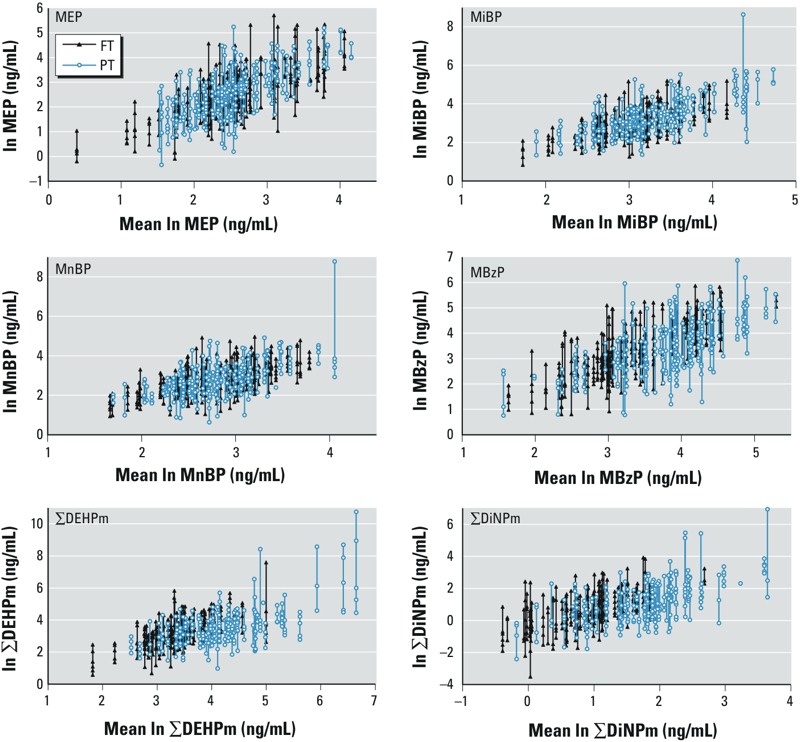
Within-subject variation of urinary phthalate metabolite levels in 58 FT infants (307 samples) from M1–M6 and 67 PT infants (290 samples) from M2–M6 for MEP, MiBP, MnBP, MBzP, ∑DEHPm, and ∑DiNPm. Vertical lines with dots represent individual samples of the same infant (*y*-axis) versus the infant’s mean phthalate metabolite level (*x*-axis). All concentrations were ln-transformed. Phthalate metabolite concentrations for PT infants at M1 were not included, because PT infants at M1 mostly were hospitalized, whereas only seven and five infants still were hospitalized at M2 and M3, respectively.

Concentrations of all metabolites were correlated, indicating that infants were simultaneously exposed to multiple phthalates. The weakest correlations were observed between MEP and MBzP, ΣDEHPm, and ΣDiNPm (parameter estimates from the standardized mixed model 0.41, 0.25, and 0.30, respectively); between MiBP and ΣDEHPm and ΣDiNPm (0.31 and 0.38, respectively); and between MnBP and ΣDEHPm and ΣDiNPm (0.36 and 0.45, respectively). Correlations among all other metabolites were stronger, with parameter estimates ranging from 0.52 (MiBP and MBzP) to 0.87 (MiBP and MnBP). From around M1 for FT and M2 for PT infants, each infant tended to stay within his or her trajectory of low or high exposure (Figure 1), although the ICC was low to moderate for most metabolites in both FT and PT infants (see Supplemental Material, Table S2). The highest ICCs were in general observed from M1 to M6 in FT infants and from M2 to M6 in PT infants indicating consistent exposure in this period, but ICCs decreased if M14 was included.

**Figure 2:**
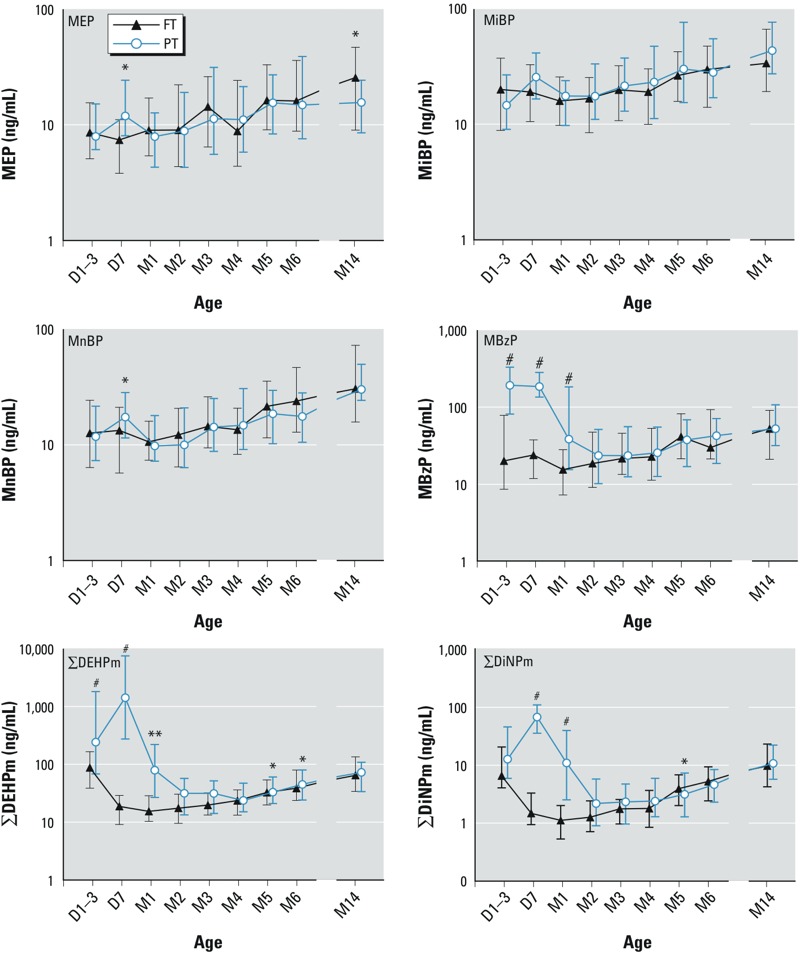
Median levels (error bars indicate the 25th–75th percentiles) of urinary phthalate metabolites in 432 samples (D1–3, *n *= 32; D7, *n *= 50; M1, *n* = 53; M2, *n* = 54; M3, *n* = 50; M4, *n* = 51; M5, *n* = 50; M6, *n* = 49; M14, *n* = 43) of FT and 462 samples (D1-3, *n* = 41; D7, *n* = 39; M1, *n* = 48; M2, *n* = 51; M3, *n* = 59; M4, *n* = 57; M5, *n* = 61; M6, *n* = 61; M14, *n* = 45) of PT infants according to age [day (D) and month (M)]. Symbols indicate statistical significance for group difference in the mixed-model analysis adjusted for weight (*p < 0.05, **p < 0.01, and ^#^*p* < 0.001).

*Factors associated with urinary phthalate metabolite levels*. Sex and breastfeeding were not significantly associated with urinary phthalate levels (see Supplemental Material, Table S3). Therefore, these factors were not included in the subsequent models. MEP, MiBP, and MnBP excreted from D1–3 to M14 showed a similar pattern for FT and PT infants except for MEP and MnBP at D7, for which PT infants had significantly higher excretion (Figure 2). Concentrations of other phthalate metabolites were significantly higher in PT infants compared with FT infants in D1–3, D7, and M1 samples for MBzP and ΣDEHPm, and in D7 and M1 samples for ΣDiNPm (Figure 2). Significantly higher concentrations of MBzP in M1 and M2 samples (*p* < 0.01), and of ΣDEHPm and ΣDiNPm in M1, M2, and M3 samples (*p* < 0.01) were estimated for hospitalized PT infants compared with PT infants at home (see Supplemental Material, Figure S1). However, differences between FT and PT infants in MBzP and ΣDEHPm (at D1–3 and D7) and ΣDiNPm (at D7 and M1) concentrations remained statistically significant even after adjustment for hospitalization (Figure 2).

After discharge from hospital, all metabolites except MBzP in FT infants increased significantly from D7 to M14 (*p* < 0.05, adjusted for weight). In discharged PT infants, a similar, significant increase was observed in all metabolites except MEP from M1 to M14 (*p <* 0.01, adjusted for weight) (see Supplemental Material, Figure S1).

*Urinary phthalate metabolites by PM age*. MBzP, ΣDEHPm, and ΣDiNPm concentrations in PT infants were significantly higher in PM weeks 30.1–34.4 and 34.1–38.0 than were concentrations in later PM weeks in both PT and FT infants (*p* < 0.01, adjusted for weight) (see Supplemental Material, Figure S2). Furthermore, concentrations of ΣDEHPm in samples collected at PM weeks 46.1–50.0 and 54.1–58.0 and of ΣDiNPm in samples collected at PM weeks 42.1–46.0 through 54.1–58.0 were significantly higher in PT compared with FT infants (see Supplemental Material, Figure S2).

**Figure 3:**
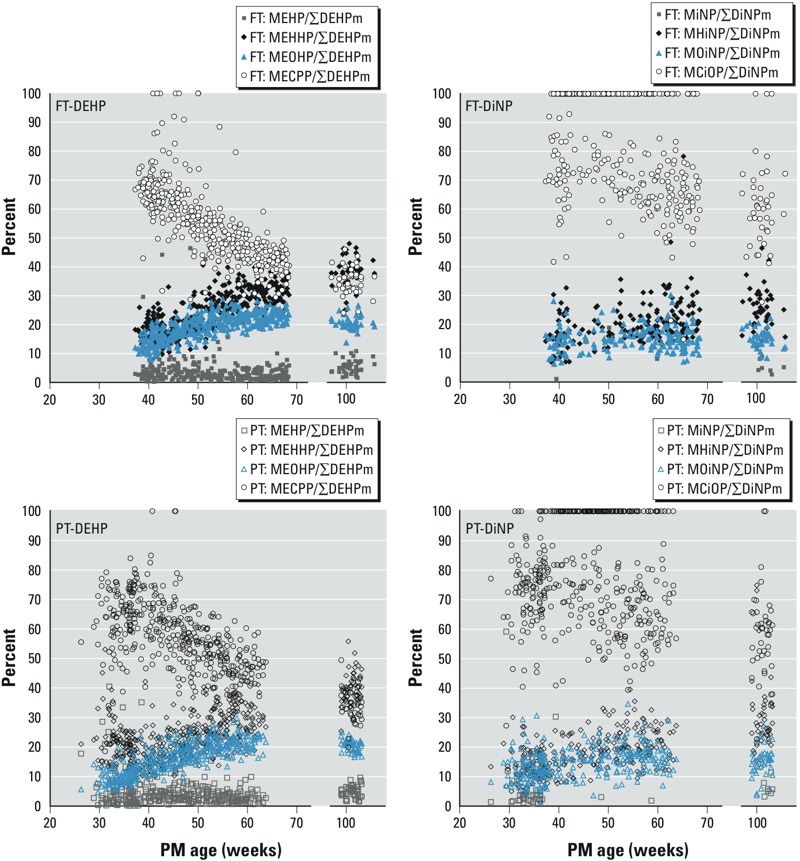
The proportion of individual DEHP or DiNP metabolites versus the total sum of all measured DEHP metabolites (∑DEHPm) or DiNP metabolites (∑DiNPm) by PM age in 58 FT (~ 432 urine samples) and 67 PT infants (~ 462 urine samples). In seven FT and three PT samples, MECPP was the only metabolite of DEHP measured (MECPP/∑DEHPm = 100%), and in 201 FT and 135 PT samples MCiOP was the only metabolite of DiNP (MCiOP/∑DiNPm = 100%) measured.

*Urinary phthalate excretion pattern*. The proportion of monoester levels (MEHP and MiNP) relative to other DEHP and DiNP metabolites, respectively, was stable during the whole follow-up (Figure 3). A considerable change in the excretion pattern of secondary metabolites was observed by increasing maturation. The relative proportion of the carboxylated metabolites decreased significantly by PM age from an average of 66% to 35% MECPP in FT and PT infants, and from 78% to 58% MCiOP in FT and from 71% to 55% MCiOP in PT infants. The proportions of other oxidized metabolites increased in the same period: hydroxylated metabolites from an average of 18% to 39% (MEHHP) and from 13% to 27% (MHiNP), and the keto-modified metabolites from 13% to 20% (MEOHP) and from 12% to 15% (MOiNP). In some samples, only carboxylated metabolites were detectable in concentrations above LODs. There was no statistically significant association of excretion patterns with estimated absolute exposure levels or sex (data not shown).

**Figure 4:**
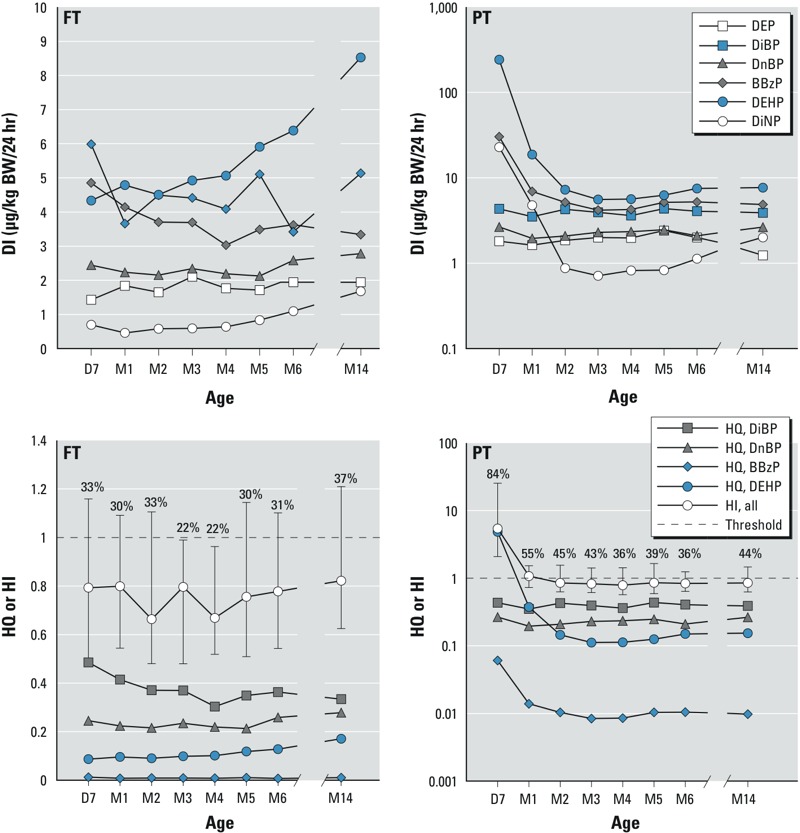
Median daily intake (DI), hazard quotient (HQ), and hazard index (HI) of phthalate diesters in FT (D7, *n* = 49; M1, *n* = 53; M2, *n* = 54; M3, *n* = 50; M4, *n* = 51; M5, *n* = 50; M6, *n* = 49; M14, *n* = 41) and PT (D7, *n* = 38; M1, *n* = 47; M2, *n* = 51; M3, *n* = 58; M4, *n* = 56; M5, *n* = 61; M6, *n* = 61; M14, *n* = 45) infants according to age [day (D) and month (M)]. BW, body weight. Median DIs are shown for the phthalate diesters (upper figures), and median levels of HQ and HI (error bars indicate the 25th–75th percentiles) are shown for DiBP, DnBP, BBzP, and DEHP (lower figures). The dashed line indicates the antiandrogenic threshold value (HI = 1), and values above error bars indicate the percentage of infants exceeding HI (HI was defined by summed ratios of estimated daily intakes to TDI based on EFSA recommendations). See Supplemental Material, Table S4, for corresponding numeric data.

*Daily intake (DI), HQ, and HI estimates*. Median estimated DIs of phthalate diesters from D7 to M14 are shown in Figure 4 (see Supplemental Material, Table S4, for corresponding numeric data). A constant estimated DI over time was observed for all phthalates, except a significant decrease of exposure to BBzP, DEHP, and DiNP in PT infants from D7 to M2–3. The median DIs of DEP, DiBP, and DnBP did not differ significantly between FT and PT infants over time. In contrast, median DIs of BBzP, DEHP, and DiNP concentrations were significantly higher in PT than in FT newborns from D7 to M2 (data not shown).

Median estimated HQs based on TDI values for DiBP, DnBP, BBzP, and DEHP are shown in Figure 4 and Supplemental Material, Table S4. At D7, > 80% of the PT infants exceeded the antiandrogenic threshold (HI > 1), whereas approximately 30% of the FT infants (and the PT infants from M2) exceeded the threshold during the entire first year of life.

## Discussion

To our knowledge, this is the first comprehensive longitudinal study of FT and PT infants from birth through 14 months of age, which documents a considerable exposure of all infants to multiple phthalates with an endocrine-disrupting potential. Our findings regarding estimated DI and accumulated HI raise concern. Most PT children and approximately one-third of FT children exceed the EFSA safety margin calculated for antiandrogenic effects, in particular shortly after birth. Such safety margins are estimated based on toxicological studies with rats defining exposure limits for either no observed effects or lowest dose with observed effect. Thus, our results raise concern because the observed phthalate exposures may put these infants at risk.

Epidemiological studies in humans have reported that phthalate exposure prenatally and in early life is associated with changes in infant sex hormones and decreased anogenital distance (AGD) (Main et al. 2006; Swan et al. 2005), allergic asthma (Bornehag and Nanberg 2010), and changes in sex-specific behavioral patterns (Miodovnik et al. 2011; Swan et al. 2010; Whyatt et al. 2012). This is corroborated by numerous animal experiments showing decreased AGD and testicular testestorone production after *in utero* exposure (Borch et al. 2006; Foster 2006; Howdeshell et al. 2007).

It has previously been shown that premature children are highly exposed to DEHP from medical equipment (Silva et al. 2006; Su et al. 2012; Weuve et al. 2006). It was assumed that this exposure was restricted to DEHP (Weuve et al. 2006). However, our study shows that higher phthalate exposure also occurred among hospitalized FT infants and was not restricted to DEHP. Although concentrations of metabolites in urine suggest that the Finnish PT infants in our population were exposed to DEP, DiBP, and DnBP at levels similar to those of the FT infants at hospital and at home, they were significantly more exposed to BBzP, DEHP, and DiNP during hospitalization compared with PT infants after discharge from hospital: The neonatal intensive care unit was the likely exposure source. After discharge, exposure patterns for BBzP, DEHP, and DiNP were similar for PT and FT infants. Regional differences may exist in the use of phthalate diesters in medical equipment. However, domestic exposure also needs to be addressed when planning preventive measures. Urinary phthalate metabolite concentrations increased with age, but the estimated DI per kilogram body weight remained constant over time. Diet has been pointed out as the main source of phthalate exposure, especially for DEHP (Wittassek et al. 2011). However, only low amounts of phthalate diesters or their metabolites have been quantified in human breast milk (Fromme et al. 2011; Main et al. 2006) but especially low-molecular phthalates such as dimethyl phthalate, DEP, and DiBP have been observed in baby care products (Sathyanarayana et al. 2008b).

It is still debated whether urinary concentrations of phthalate metabolites should be adjusted for creatinine (Lorber et al. 2011) to correct for dilution. We did not use creatinine correction for the first 3 days because levels are elevated due to maternal contamination (Matos et al. 1998). Thus, in our study, results from D1–3 were not included when analyzing data, which were creatinine corrected. Muscle mass in infants is extremely small, so creatinine-adjusted levels are extremely high. This should be kept in mind when comparing exposure levels in infants to those in older populations. During the first postnatal months, urinary dilution will show less variation due to a more consistent eating and sleeping pattern in newborns compared with older children. Creatinine correction may therefore not be as appropriate as in older populations.

Because exposure through breastfeeding is believed to be low (Fromme et al. 2011), and our study did not reveal statistically significant differences in phthalate exposure levels between breastfed and bottle-fed infants, exposure may occur through sources other than diet. The relatively constant DI in our study indicates exposure sources such as personal care products and the general home environment. Phthalates are present in many consumer products (Buckley et al. 2012; Fromme et al. 2007; Sathyanarayana et al. 2008b), they sediment in house dust (Becker et al. 2004; Langer et al. 2014), and exposure occurs through ingestion, inhalation, and dermal contact (Janjua et al. 2008; Langer et al. 2014; Wittassek et al. 2011). Because infants share their mother’s environment, exposure levels measured after birth may also reflect antenatal exposure (Sathyanarayana et al. 2008a; Wittassek et al. 2009). Both the prenatal and early postnatal periods are particularly vulnerable phases for reproductive development (Main et al. 2006; Swan et al. 2005).

Because phthalates have a short half-life in humans (Anderson et al. 2001, 2011; Koch et al. 2012), assessment of exposure levels is difficult. Concentrations of phthlate metabolites in urine are considered the best biomarker of exposure (Frederiksen et al. 2010). However, variation in urinary dilution and phthalate exposures (Sathyanarayana et al. 2008b) leads to variation in phthalate concentrations among spot urine samples collected from the same individual, which was also observed in our study. However, a given infant tended to stay within low, medium, or high levels of exposures over time, which was in accordance with previous observations (Mouritsen et al. 2013). Although we observed low-to-moderate ICCs for most phthalates, our data indicate that the within-subject variability was lower than the between-subject variability. These ICCs were similar to previous ICCs in pregnant women (Adibi et al. 2008; Frederiksen et al. 2013). In young men, ICCs were similar for MEP, MiBP, MnBP, and BBzP but higher for ΣDEHPm and ΣDiNPm (Adibi et al. 2008; Frederiksen et al. 2013). This suggests a more constant phthalate exposure in infants and pregnant women than in young men. Highest ICCs were observed from M1 to M6 in FT infants and from M2 to M6 in PT infants, indicating a relatively consistent exposure during this period. Such consistency may be created by uniform diet (breast milk and/or formula) given regularly during the waking hours and a consistent home environment during the first months of life. ICCs decreased slightly at 14 months of age, perhaps indicating more variable sources of exposure during diet transition to more solid foods and to a more active and exploratory lifestyle, including mouthing of toys and objects.

Children with a relatively high exposure to one phthalate tended also to be highly exposed to the other five phthalates, and exposures tended to be present at a relatively high level throughout infancy. Similar significant correlations were observed in several previous studies (Frederiksen et al. 2012; Koch et al. 2011; Mieritz et al. 2012; Mouritsen et al. 2013). This may be of biological significance because high simultaneous exposure may exceed a safe threshold. Our estimates of daily phthalate intakes, HQs, and HI are rough estimates and should be interpreted with caution. Urinary excretion fractions used to estimate daily intakes were derived from adult studies because they are not available for infants (Anderson et al. 2001, 2011; Koch et al. 2012). Likewise, we used estimates of the average excretion of creatinine based on 24-hr urine collections from 60 infants with gestational age 27–40 weeks and postnatal age ranging from 3–68 days (Al-Dahhan et al. 1988). Also, exposure measurements in the individual child vary over time. Thus, our data should be taken only as indicator of potential antiandrogenic effects. Thirty percent of all infants exceed the antiandrogenic threshold, and 80% of preterm babies during the first 2–3 months of life. The same children will simultaneously be exposed to other environmental chemicals (Kortenkamp and Faust 2010), which may act synergistically with phthalates. Thus, our observations raise concern.

Our study indicates a change during the first year of life in the detoxification metabolism of phthalates, which is mostly likely linked to physiological maturation processes in newborn infants. Carboxylated metabolites decreased and oxidized metabolites increased during the first year of life. A high proportion of the carboxylated metabolite MECPP has been reported before in newborns (2–5 days after birth) and preterm infants (Enke et al. 2013; Silva et al. 2006). At 14 months of age, the urinary metabolite distribution pattern for DEHP and DiNP was comparable with the pattern in older children and adults (Becker et al. 2004; Centers for Disease Control and Prevention 2013; Frederiksen et al. 2011).

Phthalates follow a two-step metabolic pathway: a phase 1 biotransformation and phase 2 conjugation. In the first step, all phthalate diesters are hydrolyzed into their respective hydrolytic monoesters. The low-molecular phthalates, such as DEP, DiBP, and DnBP, are excreted mainly in urine as free or conjugated monoesters, whereas the high-molecular monoesters such as MEHP and MiNP undergo further biotransformation to water-soluble hydroxy- or oxo-metabolites before conjugation (Koch and Calafat 2009; Rusyn et al. 2006). Phase 2 conjugation is catalyzed mainly by uridine 5´-diphosphoglucuronyl transferase (UGT) to form hydrophilic glucuronidated conjugates. An alternative pathway in high-molecular phthalates is carboxylation of the monoesters. There are about 20 UGTs, some of which are involved in glucuronidation of androgens (Alcorn and McNamara 2002), such as UGT2B17, whose expression increases significantly during the first weeks of life, whereas it is inactive in fetal liver cells or present in a low-affinity form (Coughtrie et al. 1988; Tan et al. 1990).

It has been proposed that the phthalate metabolite excretion pattern of newborns could be a consequence of prematurity or *in utero* exposure (Enke et al. 2013). However, this metabolitic pattern appeared to persist throughout the first year of life in our study population, consistent with an immature detoxification metabolism. Current risk assessments do not account for this. Because the carboxylated metabolite of DEHP has only recently become available, previous studies on DEHP exposure of newborns may have systematically underestimated exposure levels (Su et al. 2012; Weuve et al. 2006).

## Conclusions

All newborns and infants in our study population were exposed to phthalates, not only during hospitalization but also at home. Most premature babies and up to one-third of mature babies in our study population had estimated exposures that appeared to exceed current EFSA recommendations for daily intakes that were developed as guidelines to limit the risk of endocrine-disrupting effects. Our findings support the need for additional research on exposures and health effects of phthalates in infants, particularly because phthalate metabolism may be limited during the first year of life.

## Supplemental Material

(532 KB) PDF
