# Supplementary material for: A Longitudinal Study of Urinary Phthalate Excretion in 58 Full-Term and 67 Preterm Infants from Birth through 14 Months
Source: Environ Health Perspect. 2014 May 30;122(9):998–1005. doi: 10.1289/ehp.1307569 (PMC4154216; doi:10.1289/ehp.1307569)
Supplement: (532 KB) PDF [file ehp.1307569.s001.pdf]

Supplemental Material

A Longitudinal Study of Urinary Phthalate Excretion in 58 Full-Term and 67 Preterm Infants from Birth through 14 Months

Hanne Frederiksen, Tanja Kuiri-Hänninen, Katharina M. Main, Leo Dunkel, and Ulla Sankilampi

Table of Contents **Page**

Table of Contents	Page
**Table S1**	**2**
**Table S2**	**3**
**Table S3**	**4**
**Table S4**	**5**
**Figure S1**	**8**
**Figure S2**	**10**

Table S1. Creatinine adjusted urinary phthalate metabolite concentrations (ng/mg crea) in 58 full term (FT, n = 400 samples) and 67 preterm (PT, n = 419 samples) infants collected from D7 to 14 months of age.

Phthalate metabolites	FT Min.	FT 10th %ile	FT Median	FT 90th %ile	FT Max.	PT Min.	PT 10th %ile	PT Median	PT 90th %ile	PT Max.
MEP	10.0	37.7	102	393	2278	11.9	44.9	104	307	1881
MiBP	24.8	95.6	193	404	2004	38.4	107	204	761	9246
MnBP	19.6	68.7	146	295	1590	18.4	71.3	140	317	10695
MBzP	27.3	74.1	240	768	4080	21.4	84.2	302	1814	8510
MEHP			3.92	15.8	5364			6.97	95.2	40642
MEHHP		14.8	42.0	133	4359		20.6	61.9	800	51597
MEOHP		13.3	34.4	95.2	3247		17.8	44.1	388	35453
MECPP	13.2	41.4	89.7	219	13628	7.94	58.8	128	2182	229944
ΣDEHPm		103	230	616	28113		148	319	4196	410964
MiNP				1.27	19.6				4.40	1028
MHiNP				10.7	135			4.36	54.6	1241
MOiNP				7.28	114			3.47	44.3	890
MCiOP		3.73	11.1	31.9	643		5.49	16.1	244	3115
ΣDiNPm			20.3	68.6	1187			32.4	466	6534

Abbreviations: ΣDEHPm, the sum of DEHP metabolites; ΣDiNPm, the sum of DiNP metabolites.

Table S2. Intraclass correlation coefficients [ICC (95% CIs)] for ln-transformed unadjusted urinary concentrations of full term (FT) and preterm (PT) infants.

	**FT from M1 - M6**	**FT from M1 - M14**	**PT from M2 - M6**	**PT from M2 - M14**
Infants (n samples)	31 (186)	25 (175)	38 (205)	28 (168)
Phthalate metabolites				
MEP	0.48 (0.33, 0.65)	0.45 (0.29, 0.64)	0.34 (0.20, 0.51)	0.31 (0.16, 0.50)
MiBP	0.31 (0.17, 0.49)	0.36 (0.20, 0.55)	0.35 (0.21, 0.52)	0.32 (0.17, 0.52)
MnBP	0.37 (0.22, 0.55)	0.39 (0.24, 0.59)	0.23 (0.10, 0.40)	0.23 (0.09, 0.42)
MBzP	0.53 (0.38, 0.69)	0.53 (0.37, 0.71)	0.24 (0.11, 0.41)	0.33 (0.18, 0.52)
ΣDEHPm	0.39 (0.24, 0.57)	0.35 (0.20, 0.55)	0.42 (0.28, 0.59)	0.37 (0.21, 0.56)
ΣDiNPm	0.31 (0.17, 0.49)	0.29 (0.16, 0.48)	0.36 (0.21, 0.53)	0.24 (0.10, 0.43)

ICCs for PT infants at M1 were not included, because PT infants at M1 mostly were hospitalized, while only seven and five infants still were hospitalized at M2 and M3, respectively.

Table S3. Standardized parameter estimates (B) (95% confidence intervals) from the multivariate mixed linear model assessing the role of PT/FT group, time point, sex, breast feeding, hospitalization and weight in urinary phthalate metabolite secretion in 125 infants from birth to 14 months of age.

**Variable**	**MEP**	**MiBP**	**MnBP**	**MBzP**	**ΣDEHPm**	**ΣDiNPm**
PT vs. FT	0.20 (-0.07, 0.47)	-0.08 (-0.32, 0.17)	0.13 (-0.11, 0.36)	-0.14 (-0.38, 0.11)	-0.01 (-0.19, 0.17)	-0.04 (-0.2, 0.13)
Sex (male vs. female)	0.21 (-0.02, 0.44)	0.14 (-0.07, 0.35)	0.16 (-0.04, 0.35)	0.10 (-0.12, 0.36)	0.08 (-0.07, 0.23)	0.10 (-0.04, 0.24)
Weight at sampling	-0.07 (-0.16, 0.02)	-0.1 (-0.18, -0.01)	-0.07 (-0.15, 0.02)	-0.05 (-0.14, 0.03)	-0.19 (-0.26, -0.13)	-0.08 (-0.15, -0.02)
Hospitalization vs. home	0.07 (-0.18, 0.32)	0.33 (0.08, 0.59)	-0.02 (-0.28, 0.23)	-0.98 (-1.22, -0.75)	-1.48 (-1.68, -1.28)	-1.89 (-2.09, -1.68)
Breast feeding						
Complete vs. Formula	-0.15 (-0.35, 0.05)	-0.03 (-0.23, 0.16)	-0.06 (-0.25, 0.14)	0.32 (0.13, 0.50)	0.09 (-0.06, 0.24)	-0.17 (-0.32, -0.02)
Partial vs. Formula	0.05 (-0.17, 0.26)	0.11 (-0.11, 0.32)	0.09 (-0.12, 0.31)	0.24 (0.04, 0.44)	0.23 (0.06, 0.39)	0.04 (-0.13, 0.21)
Time point						
D1-3 vs. M14	-0.86 (-1.61, -0.01)	-1.27 (-2.00, -0.53)	-1.51 (-2.23, -0.79)	-1.44 (-2.13, -0.74)	-2.23 (-2.79, -1.68)	-2.2 (-2.75, -1.65)
D7 vs. M14	-0.97 (-1.71, -0.24)	-1.24 (-1.95, -0.53)	-1.40 (-2.10, -0.70)	-1.24 (-1.92, -0.57)	-2.04 (-2.58, -1.51)	-1.62 (-2.16, -1.09)
M1 vs. M14	-1.07 (-1.73, -0.40)	-1.50 (-2.15, -0.86)	-1.60 (-2.24, -0.97)	-1.42 (-2.04, -0.82)	-2.13 (-2.62, -1.64)	-1.75 (-2.24, -1.28)
M2 vs. M14	-0.91 (-1.49, -0.33)	-1.40 (-1.97, -0.84)	-1.48 (-2.03, -0.92)	-1.25 (-1.78, -0.72)	-1.9 (-2.32, -1.47)	-1.62 (-2.04, -1.20)
M3 vs. M14	-0.6 (-1.11, -0.09)	-1.06 (-1.55, -0.57)	-1.11 (-1.59, -0.62)	-1.02 (-1.49, -0.56)	-1.65 (-2.02, -1.27)	-1.47 (-1.84, -1.10)
M4 vs. M14	-0.66 (-1.10, -0.22)	-1.03 (-1.46, -0.60)	-1.04 (-1.46, -0.61)	-0.94 (-1.34, -0.54)	-1.47 (-1.79, -1.40)	-1.30 (-1.62, -0.97)

The mixed model analysis was used for statistical testing to account for the correlation structure due to the repeated measurements and multiple births in the cohort.

Table S4. Estimated daily intake, hazard quotient and hazard index of phthalate diesters in full term and preterm infants at 7 days, 2, 6 and 14 months of age.

Variable	FT Median	FT 70th %ile	FT 90th %ile	FT Max.	FT no. > threshold[a]	PT Median	PT 70th %ile	PT 90th %ile	PT Max.	PT no. > threshold[a]
**Daily intake (μg/kg/day)**										
DEP										
D7	1.43	2.05	5.24	28.5		1.81	2.94	5.72	7.78	
M2	1.65	3.33	7.27	41.2		1.86	3.30	8.44	14.0	
M6	1.94	3.13	7.12	15.1		2.00	3.42	5.69	15.3	
M14	1.94	2.77	6.95	31.0		1.24	2.64	4.22	4.83	
DiBP										
D7	4.85	5.39	8.34	15.6	3	4.35	5.62	12.2	39.0	6
M2	3.71	4.90	8.05	10.3	1	4.29	6.66	11.5	33.2	6
M6	3.63	4.35	7.15	38.9	2	4.07	5.45	23.6	179	11
M14	3.34	4.66	8.11	13.4	2	3.90	6.51	13.8	28.1	7
DnBP										
D7	2.45	3.32	5.39	7.67	0	2.66	3.64	5.13	12.0	1
M2	2.15	3.27	5.59	7.30	0	2.09	2.75	5.02	7.57	0
M6	2.59	3.33	4.70	25.9	2	2.10	2.93	5.76	174	3
M14	2.78	3.35	5.41	7.81	0	2.65	3.21	7.46	17.3	3
BBzP										
D7	5.99	8.05	12.8	21.7	0	30.4	38.1	66.5	108	0
M2	4.49	7.50	14.7	21.4	0	5.18	7.54	29.0	52.3	0
M6	3.42	7.05	21.1	34.2	0	5.22	7.89	16.9	39.1	0
M14	5.14	6.53	23.5	74.1	0	4.88	11.9	32.1	68.5	0
DEHP										
D7	4.34	6.88	12.9	56.8	1	243	1162	3267	7127	30
M2	4.51	7.04	11.9	37.4	0	7.27	11.6	160	9970	6
M6	6.39	9.41	16.6	40.0	0	7.51	9.67	19.3	179	1
M14	8.53	10.8	21.5	62.6	1	7.68	11.4	20.3	39.0	0

Variable	FT Median	FT 70th %ile	FT 90th %ile	FT Max.	FT no. > threshold[a]	PT Median	PT 70th %ile	PT 90th %ile	PT Max.	PT no. > threshold[a]
DiNP										
D7	0.70	1.17	3.33	18.7		22.9	29.6	41.4	61.3	
M2	0.58	0.83	1.32	2.33		0.88	1.74	22.4	240	
M6	1.10	1.73	4.03	7.06		1.13	1.56	3.39	14.0	
M14	1.68	2.81	5.77	11.6		2.01	3.21	7.66	21.8	
**Hazard quotient (HQ)**										
DiBP[b]										
D7	0.49	0.54	0.83	1.56	3	0.43	0.56	1.22	3.90	6
M2	0.37	0.49	0.81	1.03	1	0.43	0.67	1.15	3.32	6
M6	0.36	0.43	0.72	3.89	2	0.41	0.54	2.36	17.95	11
M14	0.33	0.47	0.81	1.34	2	0.39	0.65	1.38	2.81	7
DnBP										
D7	0.24	0.33	0.54	0.77	0	0.27	0.36	0.51	1.20	1
M2	0.22	0.33	0.56	0.73	0	0.21	0.28	0.50	0.76	0
M6	0.26	0.33	0.47	2.59	2	0.21	0.29	0.58	17.4	3
M14	0.28	0.34	0.54	0.78	0	0.26	0.32	0.75	1.73	3
BBzP										
D7	0.01	0.02	0.03	0.04	0	0.06	0.08	0.13	0.22	0
M2	0.01	0.02	0.03	0.04	0	0.01	0.02	0.06	0.10	0
M6	0.01	0.01	0.04	0.07	0	0.01	0.02	0.03	0.08	0
M14	0.01	0.01	0.05	0.15	0	0.01	0.02	0.06	0.14	0
DEHP										
D7	0.09	0.14	0.26	1.14	1	4.85	23.2	65.3	143	30
M2	0.09	0.14	0.24	0.75	0	0.15	0.23	3.21	199	6
M6	0.13	0.19	0.33	0.80	0	0.15	0.19	0.39	3.57	1
M14	0.17	0.22	0.43	1.25	1	0.15	0.23	0.41	0.78	0

Variable	FT Median	FT 70th %ile	FT 90th %ile	FT Max.	FT no. > threshold[a]	PT Median	PT 70th %ile	PT 90th %ile	PT Max.	PT no. > threshold[a]
**Hazard Index (HI)**										
D7	0.79	1.04	1.69	2.29	16	5.44	24.3	66.2	143	32
M2	0.66	1.03	1.56	2.11	18	0.85	1.42	5.07	200	23
M6	0.78	1.00	1.52	6.82	15	0.84	1.09	3.59	38.9	22
M14	0.82	1.13	1.61	3.26	15	0.85	1.38	2.25	4.48	22

HI = sum of HQs. HQ and HI were based on EFSA´s tolerable daily intake (TDI) for DnBP (10 µg/kg/day), BBzP (500 µg/kg/day) and DEHP (50 µg/kg/day).

[a]Numbers of FT or PT infants with DI > TDI (for DI estimates), HQ > 1 (for HQ estimates), or HI > 1 (for HI estimates). [b]TDI for DnBP was used to calculate HQ for DiBP. FT: n=49 (D7), n=54 (M2), n=49 (M6), n=41 (M14). PT: n=38 (D7), n=51 (M2), n=61 (M6), n=45 (M14).

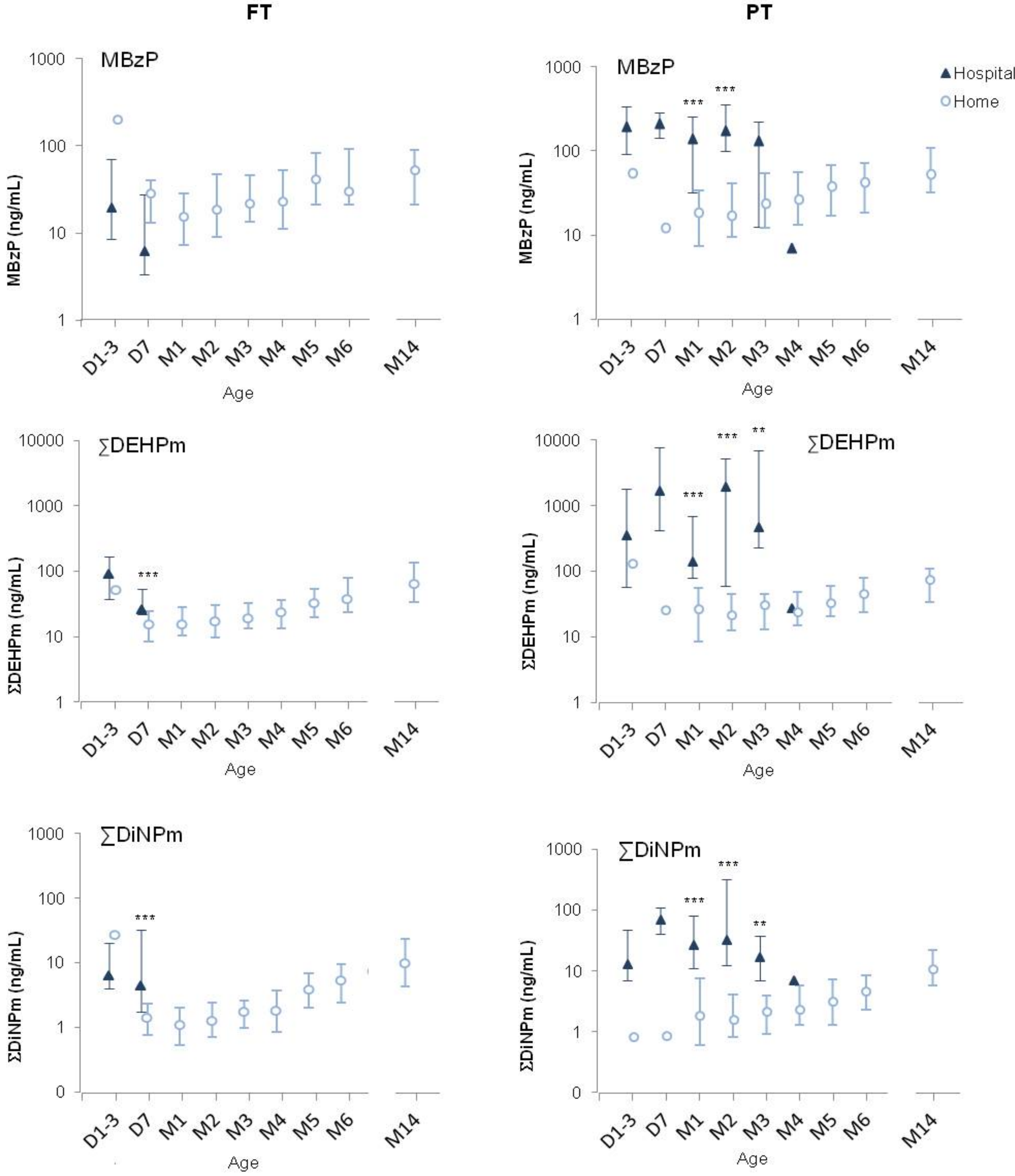

Figure S1. Comparison of MBzP, ∑DEHPm and ∑DiNPm levels in FT and PT infants at hospital (solid triangles) or at home (open circles) from the age of day (D) 1-3 to the corrected age of 14 months (M14). Median levels (error bars indicate the 25th – 75th percentiles) in 432 samples of full term (FT) and 462 samples of preterm (PT) are shown. FT at hospital: D1-3, n=31; D7, n=9; FT at home: D1-3, n=1; D7, n=41 M1, n=53; M2, n=54; M3, n=50; M4, n=51; M5, n=50; M6, n=49; M14, n=43; PT at hospital: D1-3,

n=40; D7, n=37; M1, n=27; M2, n=7; M3, n=5; M4, n=1; PT at home: D1-3, n=1; D7, n=2; M1, n=21; M2, n=44; M3, n=54; M4, n=56; M5, n=61; M6, n=61; M14, n=45. The asterisks indicate statistical significance for difference between infants at home or hospital in the mixed-model analysis performed separately for FT and PT infants and adjusted for actual weight (** $p < 0.01$; *** $p < 0.001$).

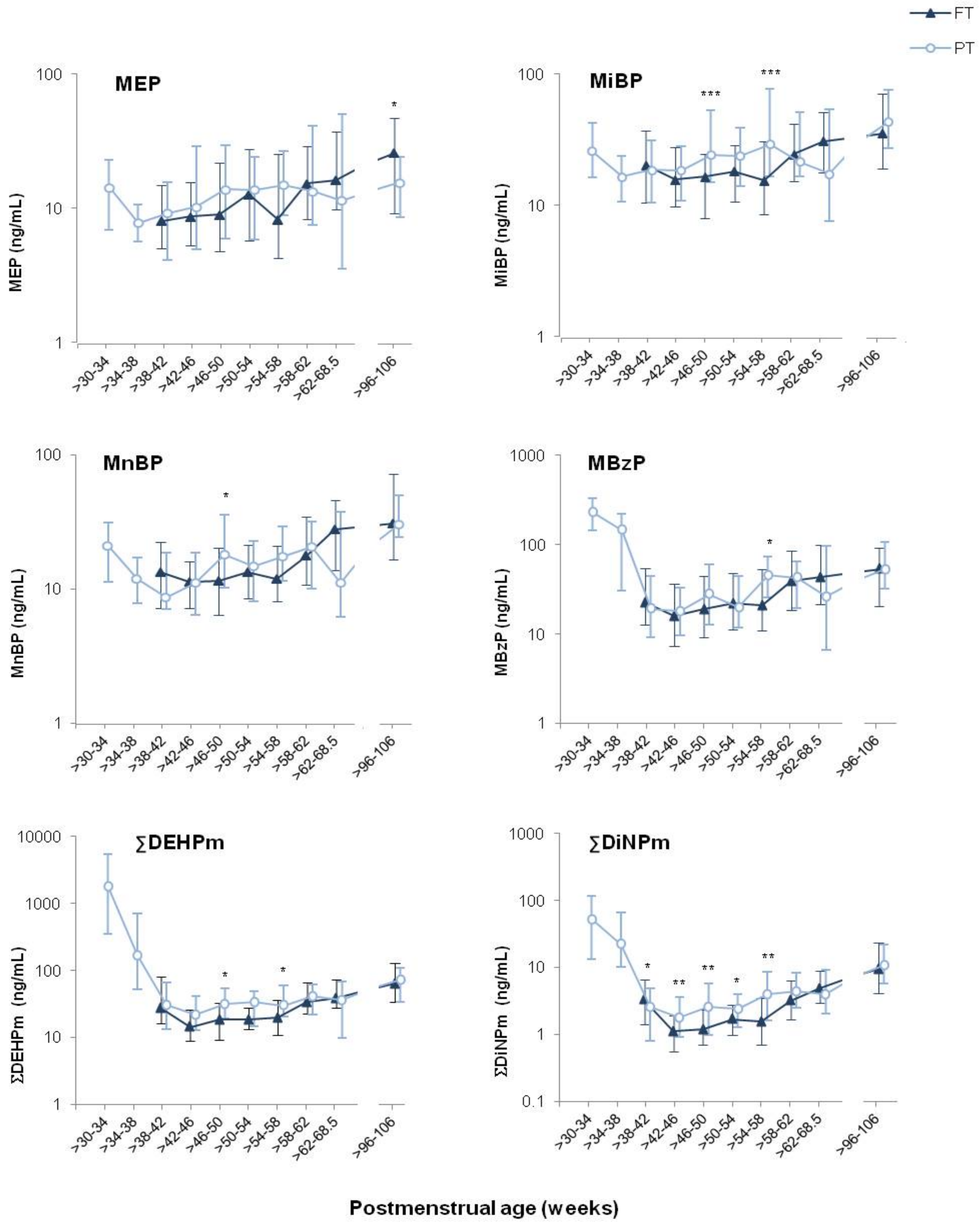

Figure S2. Median levels (error bars indicate the 25th – 75th percentiles) of urinary phthalate metabolites in 403 samples (w>38-42, n=72; w>42-46, n=62; w>46-50, n=47; w>50-54, n=44; w>54-58, n=43;

w>58-62, n=43; w>62-68.4, n=46; w>96-106, n=42) of full term (FT) and 452 samples (w>30-34, n=42; w>34-38, n=68; w>38-42, n=44; w>42-46, n=50; w>46-50, n=59; w>50-54, n=49; w>54-58, n=51; w>58-62, n=35; w>62-68.5, n=9; w>96-106, n=45) of preterm (PT) infants according to postmenstrual age categories (weeks (w)). The asterisks indicate statistical significance for difference between PT and FT infants in the mixed-model analysis after adjustment for weight at sampling (*$p < 0.05$ and **$p < 0.01$ and ***$p < 0.001$).
